# Evaluation of the effect of sleeve gastrectomy versus Roux-en-Y gastric bypass in patients with morbid obesity: multicenter comparative study

**DOI:** 10.1007/s00423-024-03341-9

**Published:** 2024-05-10

**Authors:** Omar Thaher, Friederike Wollenhaupt, Roland S. Croner, Martin Hukauf, Christine Stroh

**Affiliations:** 1 Department of Surgery, Marien Hospital Herne, Ruhr-Universität Bochum, Hölkeskampring 40, 44625 Herne, Germany; 2 Department for Pediatrics and Adolescent Medicine, Asklepios Klinikum Heidberg, Tangstedter Landstraße 400, 22417 Hamburg, Germany; 3 Department of General, Visceral, Vascular and Transplant Surgery, University Hospital Magdeburg, Leipziger Str. 44, 39120 Magdeburg, Germany; 4 StatConsult Society for Clinical and Health Services Research mbH, Am Fuchsberg 11, 39112 Magdeburg, Germany; 5 Department of General, Abdominal and Pediatric Surgery, Municipal Hospital, Straße Des Friedens 122, 07548 Gera, Germany

**Keywords:** Sleeve gastrectomy, Gastric bypass, Bariatric surgery, Morbidity and mortality, Complications

## Abstract

**Introduction:**

Roux-en-Y gastric bypass (RYGB) and sleeve gastrectomy (SG) are the two most performed techniques in bariatric surgery. The aim of this study is to compare two surgical procedures in terms of weight loss and the development of comorbidities such as type II diabetes mellitus T2D, arterial hypertension, sleep apnea (OSAS), and gastroesophageal reflux disease (GERD).

**Methods:**

Data from the German Bariatric Surgery Registry (GBSR) from 2005 to 2021 were used. 1,392 RYGB and 1,132 SG primary surgery patients were included. Minimum age 18 years; five-year follow-up data available. Tests were performed with a 5% significance level.

**Results:**

Loss of follow-up 95.41% within five years. Five years after surgery, the RYGB showed significant advantages in terms of excess weight loss (%EWL 64.2% vs. 56.9%) and remission rates of the studied comorbidities: hypertension (54.4% vs. 47.8%), OSAS (64.5% vs. 50.1%), and GERD (86.1% vs. 66.9%). Compared to the pre-test, individuals diagnosed with insulin-dependent T2D showed significant improvements with RYGB over a five-year period (remission rate: 75% vs. 63%). In contrast, non-insulin-dependent T2D showed no significant difference between the two approaches (p = 0.125).

**Conclusion:**

Both surgical procedures resulted in significant weight loss and improved comorbidities. However, the improvement in comorbidities was significantly greater in patients who underwent RYGB than in those who underwent SG, suggesting that the RYGB technique is preferable.

Nevertheless, RYGB requires a high degree of surgical skill. Therefore, acquiring expertise in the technical facets of the surgery is essential to achieving favorable outcomes.

## Introduction

Severe obesity is associated with a variety of chronic conditions that compromise overall health and increase the risk of death in obese individuals [1, 2]. An increase in BMI significantly increases the likelihood of developing obesity-related conditions such as arterial hypertension aHTN, diabetes mellitus type 2 (T2D), and apnea (OSAS) [3, 4]. Most non-surgical treatments have shown limited effectiveness in reducing weight and managing obesity-related comorbidities [5]. Clinical studies have consistently shown that bariatric surgery is an effective method for weight loss and management of obesity-related comorbidities [6]. Bariatric surgery is a growing surgical field focused on the treatment of obesity due to the rapid increase in weight worldwide [7].

Surgical techniques have historically been classified as malabsorptive, restrictive, or mixed. However, the efficacy of different bariatric surgical approaches in terms of weight loss, comorbidity reduction, and perioperative outcomes varies widely. Sleeve gastrectomy (SG) and Roux-Y gastric bypass (RYGB) have long been recognized as standard bariatric surgical procedures [8].

RYGB and SG each have their own advantages and disadvantages. SG has experienced a rapid rise as a bariatric surgical procedure for patients with obesity due to its technical simplicity [9]. However, when it comes to treating obesity-related conditions, bariatric surgery has shown more favorable results when using techniques like RYGB as opposed to SG [10].

The objectives of this study are to compare the efficacy of SG and RYGB in achieving remission of obesity-related comorbidities, to identify any perioperative complications that may be associated with both procedures, and to provide an update on weight status five years after surgery. By comparing the results of our study with those of international randomized trials, we hope to provide an overview of the current status and outcomes of both procedures in Germany and draw comparisons between the outcomes in Germany and those of international randomized trials.

## Material and methods

### Data collection and study design

The study was conducted in accordance with the principles specified in the Declaration of Helsinki, which governs biomedical research. All participants completed an informed consent form before data were entered into the registry. Patients were included in the study if they consented to follow-up.

Data were collected online for the purpose of quality assurance research. The research involved entering the relevant data into a specially designed database. StatConsult performed the data analysis using export data from 04/2021, which were reviewed, and plausibility checked by the study director.

**Fig. 1:**
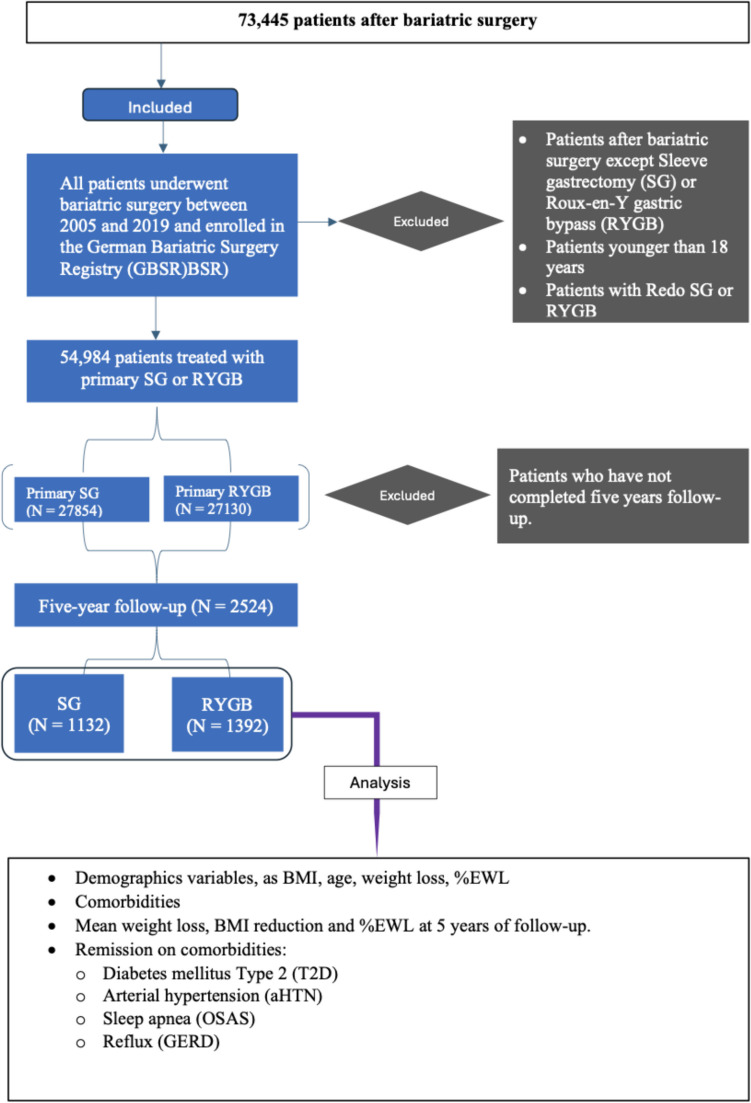
Flow chart of the study's and patients' criteria

The current retrospective multicenter analysis of the German Bariatric Surgery Registry (GBSR) database, which was compiled prospectively between January 2005 and April 2021, used the following inclusion criteria (Fig. 1):

- Minimum age 18 years.
- RYGB or SG is acceptable as the primary surgical procedure.
- A valid five-year follow-up (1,643–2,008 days after surgery) is required.

Selection of the appropriate surgical procedure was influenced by individual patient characteristics and physician indications. Data collected included:

- Population statistics, weight data, comorbidities, and ASA classification.
- Long-term follow-up data, including weight development, BMI reduction, %EWL, development of T2D, aHTN, OSAS, and GERD.
- Patients were considered to be completely cured of reflux at follow-up examinations if the symptoms and medication treatment of reflux were no longer present after surgery. Some clinics added pH monitoring and endoscopy; however, this was not a universal practice and not a standard procedure to rule out reflux disease after bariatric surgery.
- The development of DM, aHTN, and OSAS was compared with the original preoperative data based on medication and symptom development after surgery. Different categories were analyzed for all comorbidities compared: Worsening, improvement, new development, no change or complete remission of comorbidities.

### Statistical analysis

Statistical analyses were performed using SAS 9.4 at a 5% significance level, with a p-value of less than or equal to 0.05 indicating a statistically significant difference.

#### Descriptive and univariate/unadjusted analyses:

Analysis included examination of mean, standard deviation, and number of patients for quasi-continuous variables and root-transformed data. The mean values of the original and transformed data differ. Continuous variables are reported as minimum, lower quartile, median, upper quartile, and maximum and a paired t-test is used for comparison.

Unadjusted analyses are used to analyze the effect of a single variable on a target parameter, with a focus on comparing surgical procedures. The chi-squared test is used for categorical outcome variables, and the Satterthwaite robust t-test is used for continuous outcome variables. Significant deviations from the normal distribution require a root function transformation to approximate the distribution.

## Results

The study included a total of 73,445 patients. Of these, 54,984 patients underwent either RYGB or SG. Of the 2,524 patients who were followed up for five years, 55.2% (n = 1,392) underwent RYGB, whereas 44.8% (n = 1,132) underwent SG. The majority of both procedures were performed using laparoscopic techniques (> 97%), and SG had a significantly shorter operating time. Postoperative and total hospital stay did not show a significant advantage for either group.

The study found a significant gender distribution among the 2,524 patients, with a higher proportion of females (75.2%) than males (24.8%). Among the SG group, 357 (31.5%) patients were male and 775 (68.5%) were female. In the RYGB cohort, 19.3% were male and 80.7% were female (p < 0.001).

**Fig. 2:**
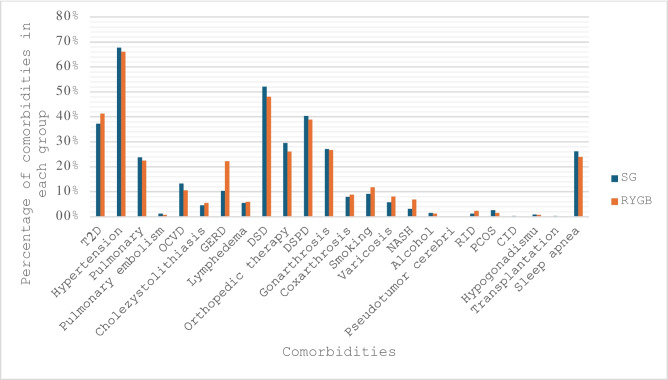
Distribution of comorbidities at the time of surgery. OCVD = other cardiovascular disease, DSD = degenerative skeletal disease, DSPD = degenerative spinal disease, NASH = nonalcoholic steatohepatitis, RID = rheumatoid inflammatory disease, PCOS = polycystic ovary syndrome, CID = chronic inflammatory bowel disease.

Of the total of 2,339 patients, 92.7% had a documented comorbidity at the time of surgery (Fig. 2). According to the study, there was considerable variation in patients’ comorbidities. Cardiovascular disease and degenerative skeletal disease were more common in SG patients, whereas smoking, varicosis, T2D, insulin-dependent diabetes mellitus (IDDM), GERD, and non-alcoholic steatohepatitis (NASH) were more common in RYGB patients (p < 5% overall). Table 1 and Fig. 2 provide a brief summary of the comorbidities identified preoperatively and assessed during the follow-up period.

**Table 1:** Demographic distribution of age, height, body weight, BMI, and associated diseases studied in the long-term follow-up

		Surgical procedure										*p* value
		SG					RYGB					
		N	%	Min	Max	Mean ± STD	N	%	Min	Max	Mean ± STD	
Age (years)		1132		18	73	44.9 ± 11.5	1392		18	74	45.0 ± 10.8	0.761
Height (cm)		1132		124	201	170.2 ± 9.8	1391		141	204	168.8 ± 9.0	**< 0.001**
Weight (kg)		1132		77	293	146.4 ± 30.5	1391		77	238	136.8 ± 23.4	**< 0.001**
BMI (kg/m^2^)		1132		26.6	103.4	50.4 ± 8.9	1391		32.7	90.5	47.9 ± 6.8	** < 0.001**
ASA ^	ASA I	45	4.0				40	2.9				**< 0.001**
	ASA II	444	39.3				648	46.6				
	ASA III	614	54.3				682	49.1				
	ASA IV	28	2.5				20	1.4				
Comorbidities (total)		1056	93.3				1283	92.2				0.284
T2DM		379	37.2				550	41.3				**0.044**
IDDM		115	11.3				188	14.1				**0.043**
NIDDM		221	21.7				323	24.2				0.144
aHTN		766	67.7				919	66.0				0.382
OSAS		297	26.2				333	23.9				0.182
GERD		118	10.4				309	22.2				** < 0.001**

The analysis showed no significant difference in age distribution between the SG and RYGB groups. Patients treated with SG had a higher level of morbidity and a higher body mass index (BMI). During the first five years after surgery, the mean BMI decreased from 50.4 kg/m^2^ to 36.1 kg/m^2^ (SG) and from 47.9 kg/m^2^ to 33.6 kg/m^2^ (RYGB) with no statistically significant difference between the two groups. However, RYGB resulted in a significantly higher %EWL (p < 0.001) (Tables 1 and 2).

**Table 2:** Overview of weight loss, BMI reduction, and %EWL after 5 years

	SG			RYGB			p-value
	Min	Max	Mean ± STD	Min	Max	Mean ± STD	
Weight loss (kg)	-14	141	41.5 ± 21.7	-19	117	40.7 ± 17.1	0.292
BMI-Reduction (kg/m^2^)	-6.2	69.0	14.3 ± 7.3	-7	55.7	14.3 ± 5.9	0.968
%EWL	-36.9	273.7	57.9 ± 25.2	-88.7	140.8	64.2 ± 24.0	**< 0.001**

Five years after surgery, both SG and RYGB showed improvements in the recorded comorbidities with complete and partial remissions. Table 3 shows a summary of the change in comorbidities in each group.

**Table 3:** Comparison of the development of comorbidities in both groups after 5 years, relative to the time of surgery

		SG		RYGB		p- value
		N	%	N	%	
IDDM	Yes ➜ No	80	69.6	143	76.1	0.315
	Yes ➜ Yes	35	30.4	45	23.9	
	De-Novo	5	0.49*	13	1.1*	
NIDDM	Yes ➜ No	185	83.7	278	86.3	0.698
	Yes ➜ Yes	36	16.3	44	13.7	
	De-Novo	9	1.0*	13	0.84*	
aHTN	Yes ➜ No	366	47.8	499	54.4	**0.024**
	Yes ➜ Yes	400	52.2	418	45.6	
	De-Novo	14	3.8*	19	4.0*	
OSAS	Yes ➜ No	149	50.1	214	64.5	**0.001**
	Yes ➜ Yes	148	49.9	118	35.5	
	De-Novo	12	1.4*	14	1.3*	
GERD	Yes ➜ No	79	66.9	266	86.1	** < 0.001**
	Yes ➜ Yes	39	33.1	43	13.9	
	De-Novo	299	29.5*	108	10.0*	

The percentages for the yes—no and yes—yes categories refer to the number of patients with the respective comorbidity at the time of surgery and after 5 years

^*^The percentages of the de-novo category refer to the patients without the respective comorbidity at the time of surgery and with this comorbidity after 5 years, but the p-value includes all three categories

When examining the progression of comorbidities from the time of surgery to five years postoperatively, RYGB showed a significant advantage in aHTN, OSAS, and GERD (Table 3). In addition, RYGB showed a significant advantage in IDDM when comparing the situation five years after surgery with the most recent examination (Table 4).

**Table 4:** Descriptive statistics: change in comorbidities at 5-year follow-up (changes refer to the time point postoperatively and the last previous visit(s) and thus not directly to the baseline value)

Change in comorbidity		Surgical procedure				
		SG		RYGB		
		n	%	n	%	p
IDDM	Reduction	6	6.5	23	16.0	**0.003**
	Worsening	2	2.2	0	0	
	No change	28	30.4	20	13.9	
	No more therapy required	52	56.5	85	59.0	
	NIDDM – > IDDM	2	2.2	5	3.5	
	De-novo	2	2.2	11	7.6	
NIDDM	Reduction	11	6.8	11	4.9	0.125
	Worsening	3	1.9	4	1.8	
	No change	28	17.3	26	11.6	
	No more therapy required	115	71.0	164	73.2	
	IDDM – > NIDDM	0	0	0	0	
	De-novo	5	3.1	19	8.5	
aHTN	Reduction	108	15.1	138	17.3	**0.018**
	Worsening	11	1.5	9	1.1	
	No change	253	35.5	224	28.1	
	No more therapy required	299	41.9	360	45.2	
	De-novo	42	5.9	66	8.3	
OSAS	Reduction	57	20.3	78	26.3	**< 0.001**
	Worsening	102	36.3	53	17.8	
	No change	0	0	0	0	
	No more therapy required	121	43.1	165	55.6	
	De-novo	1	0.4	1	0.3	
GERD	Reduction	59	15.6	58	31.9	**< 0.001**
	Worsening	120	31.8	25	13.7	
	No change	0	0	0	0	
	No more therapy required	111	29.4	56	30.8	
	De-novo	87	23.1	43	23.6	

Regarding postoperative de-novo of comorbidities, IDDM, NIDDM, aHTN, and OSAS did not differ between the two surgical procedures. However, there was a significant difference in de-novo postoperative GERD, which was almost three times more frequent after SG, but also occurred in 10% after RYGB (Table 4).

## Discussion

Bariatric and metabolic surgery has been empirically shown to be more effective in achieving weight loss than non-surgical approaches [18, 19]. Patients who underwent bariatric surgery experienced a significant weight loss, with a mean reduction of 26 kg, according to a meta-analysis [20]. This finding contrasts with the results of non-surgical treatments. However, a comprehensive risk assessment is essential, especially for elderly and critically ill patients [21].

The present analysis analyzes patients who were enrolled in the registry study after undergoing primary SG or RYGB, with a follow-up period of five years.

The patient’s condition and the surgical center’s expertise together determined the surgical indication and procedure selection.

Particularly in regions such as the Middle East and the United States, where chronic obesity is widespread, the current prevalence of SG is approximately 70% [11]. This pattern is also observed in Germany and Europe [12]. In an international comparison, our data suggest that the indication for surgery in Germany is relatively late and occurs only in the transition from obesity to superobesity (BMI > 50 kg/m^2^). The mean BMI in this study was 49 kg/m^2^.

Due to a lack of follow-up, the study was able to evaluate only 2,524 of the 54,984 patients for whom data were available at five years postoperatively. The decrease in follow-up rates (95.41%) over a 5-year period is due to the patients’ commitment to attending follow-up sessions rather than being influenced by the treating clinics. The substantial loss to follow-up rate implies that long-term outcomes may exceed those of the “lost” residual population; therefore, a thorough critical evaluation of the long-term data is imperative.

The analysis yielded notable results due to the variation within each category and the gender disparities in the distribution of bariatric surgery.

We believe that the consistent use of SG in male patients results in a higher preoperative BMI compared to female patients. Other studies that have examined patients with preoperative elevated BMIs have reached the same conclusion: SG was used at a higher rate compared to RYGB [13, 14].

Regarding the reduction in BMI, the %EWL, and weight loss after both procedures, three studies [15–17] demonstrated a greater reduction in BMI after RYGB, while two studies [18, 19] found no significant difference (Table 5). Eight studies [15–22] used %EWL as a measure of weight loss after both procedures. Three studies [15, 16, 20] reported a significantly higher %EWL five years after RYGB (Table 6). Our research findings indicate that RYGB demonstrated a statistically significant superiority in terms of %EWL. However, there were no significant differences seen in weight loss or change in BMI between the two procedures (Tables 2, 5, and 6). Based on the development of BMI, weight and %EWL and the preoperative values, the statement about %EWL development in our study should be viewed skeptically due to the different initial weight of the two groups. Therefore, this progress should not be the only consideration when deciding on surgical procedures; other demographic and patient-specific factors should also be taken into account. In addition, the initial BMI and perioperative conditions of the patients may have influenced the choice of procedure but not the postoperative course. In our opinion, the surgical procedure has a greater impact on weight loss, %EWL, and BMI decrease, as indicated in the literature and our study results, rather than the initial preoperative weight and BMI values, despite statistical significance.

**Table 5:** Prospective studies with comparative results on development of BMI between SG and RYGB at baseline and 5-year follow-up and comparison with the results of our study

Author	BMI Baseline (kg/m^2^) + STD		BMI at five years follow-up		BMI Reduction (kg/m^2^)		p-value at 5 years follow-up
	SG	RYGB	SG	RYGB	SG	RYGB	
Zhang et al. [16]	38.5 ± 4.2	39.3 ± 3.8	32.2 ± 4.4	29.8 ± 3.7	6.3	9.5	**p = 0.02**
Schauer et al. [17]	36.0	37.0	29.3	28.9	6.7	8.1	**p = 0.02**
Peterli et al. [18]	43.5	44.3	32.5	31.6	11.0	12.7	p = 0.29
Salminen et al. [19]	47.3	48.4	36.5	35.4	10.8	13.0	p = 0.54
Toolabi et al. [15]	40.0 ± 5.8	47.0 ± 7.3	32.3 ± 0.5	29.5 ± 0.5	7.7	17.5	**p = 0.002**
GBSR	50.4 ± 8.9	47.9 ± 6.8	36.1	33.6	14.3 ± 7.3	14.3 ± 5.9	p = 0.968

**Table 6:** Prospective studies with comparative results on %EWL and weight between SG and RYGB 5-year follow-up and comparison with the results of our study

Author	Parameters	SG	RYGB	p-value
Zhang et al. [16]	%EWL	63.2 ± 24.5	76.2 ± 21.7	**p = 0.02**
Leyba et al. [21]	%EWL	67.3	69.8	p > 0.05
Ignat et al. [20]	%EWL	65.1	74.8	**p = 0.045**
Perrone et al. [22]	%EWL	70.3	72.3	p > 0.05
Schauer et al. [17]	- kg	18.6	23.2	**p = 0.01**
Peterli et al. [18]	- kg	33.0	36.6	p = 0.19
Salminen et al. [19]	%EWL	49.0	57.0	p > 0.05
Toolabi et al. [15]	%EWL	61.9	79.4	**p = 0.001**
GBSR	%EWL - kg	57.9 ± 25.2 41.5 ± 21.7	64.2 ± 24.0 40.7 ± 17.1	**p < 0.001** p = 0.292

In the current study, more than 90% of patients had preoperative comorbidities. However, the follow-up period was only five years and focused on T2D, aHTN, OSAS, and GERD.

According to the available studies, there were no significant changes between the two groups five years after surgery, even though both surgeries improve T2D [15–17]. Perrone et al. [22] found a significant difference in T2D remission rate at five years in favor of RYGB. 9,710 patients with T2D were followed for five years in a large American registry study [23]. The study reported first-year remission rates for RYGB (59.2%) and SG (55.7%). There were significant differences between the two groups at the five-year mark: remission rates increased to 86.1% after RYGB and 83.5% after SG. When comparing the preoperative and five-year follow-up status for remission of IDDM and NIDDM, our results did not show a statistically significant difference. There was a significant difference in the remission of IDDM in favor of the RYGB group five years after surgery compared to the previous controls during follow-up (p = 0.003), but not in the remission of NIDDM (p = 0.125). Therefore, the results of the literature and our registry study support the hypothesis that malabsorptive bariatric procedures (RYGB, BPD/DS) are more effective than restrictive procedures (SG, gastric banding) in terms of T2D remission.

In the case of aHTN, Salminen et al. [19] found that the remission rate after RYGB was significantly higher than after SG. Other studies [15–18, 21, 22] have found only a trend towards a benefit for RYGB compared to SG, but no significant benefit was observed. Our GBSR study showed remarkable remission rates for aHTN five years after the above surgical procedures (Table 7). In contrast to the remission rate of 47.8% for SG, RYGB resulted in a significantly higher remission rate of 54.4% (p = 0.024) when comparing the two surgical procedures.

**Table 7:** Prospective studies with comparative results on comorbidity remission between SG and RYGB at 5-year follow-up and comparison with the results of our study

	Author		Remission criterion	SG (%)	RYGB (%)	p-value (Significance)
T2D	Zhang et al. [16]			88.9%	87.5%	NS
	Leyba et al. [21]		HbA1_c_ < 6%	100%	66.6%	p > 0.05
	Perrone et al. [22]		Medication	14.6%	33.3%	**p = 0.03**
	Schauer et al. [17]		HbA1_c_ < 6%	23.4%	28.6%	p = 0.53
	Peterli et al. [18]		Medication	76.9%	75.0%	p > 0.99
	Salminen et al. [19]		Medication	12.0%	25.0%	p > 0.99
	Toolabi et al. [15]		Medication	77.0%	82.0%	NS
	GBSR	IDDM	Medication	69.6%	76.1%	p = 0.315
		NIDDM		83.7%	86.3%	p = 0.698
Hypertension	Zhang et al. [16]		Medication	60.0%	66.7%	NS
	Leyba et al. [21]		Medication	100%	100%	p > 0.05
	Perrone et al. [22]		Medication	27.8%	36.6%	NS
	Schauer et al. [17]		RR (mmHg)	- 8.3 Syst - 8.1 Dias	- 3.3 Syst - 5.8 Dias	SBP: p = 0.78 DBP: p = 0.86
	Peterli et al. [18]		Medication	87.5%	92.2%	p > 0.99
	Salminen et al. [19]		Medication	64.7%	80.8%	**p = 0.02**
	Toolabi et al. [15]		Medication	49.0%	55.0%	NS
	GBSR		Medication	47.8%	54.4%	**p = 0.024**
Sleep apnea	Zhang et al. [16]		Therapy	100%	100%	NS
	Leyba et al. [21]		Therapy	-	100%	NS
	Perrone et al. [22]		Therapy	18.1%	23.7%	NS
	Peterli et al. [18]		Therapy	95.8%	95.4%	p > 0.99
	GBSR		Therapy	50.1%	64.5%	**p = 0.001**
GERD	Peterli et al. [18]		Medication, Symptoms	34.1%	66.7%	**p = 0.02**
	GBSR		Medication, Symptoms	66.9%	86.1%	**p < 0.001**

NS = not significant. SBP = systolic blood pressure, DBP = diastolic blood pressure

Comparing the two surgical groups, slightly more than one fifth of the patients in our study had OSAS (Table 1). Both cohorts had significant remission rates. They were significantly higher after RYGB with 64.5% compared to 50.1% after SG (Table 3). The present study compared four searches [16, 18, 21, 22], all of which examined the progression of OSAS (Table 7). Remission rates were found to be predominantly high for both procedures, with no significant differences. Consistent with the results of the current study and the existing literature, both procedures induce OSAS remission. We believe that the differences between the two procedures do not only depend on the surgical approach, but also on demographic variables, postoperative weight loss, and observation schedule.

It has been observed that GERD is more common after SG than after RYGB [24]. Some surgeons even require preoperative impedance and manometry measurements. If positive results are obtained, RYGB [25] should be performed. Significant determinants associated with the development of gastroesophageal reflux disease (GERD) after SG are fundus resection, suspension apparatus modifications at the gastroesophageal junction, and the high-pressure system of the sleeve stomach [26, 27]. As a result, the incidence of de-novo GERD is high, while the remission rate of pre-existing GERD is minimal [25, 28]. In our study, 10% of cases undergoing RYGB developed de-novo GERD, compared to 29.5% in SG. Peterli et al. [18] observed comparable results in their study of de-novo GERD: 31.6% adherence to SG and 10.7% adherence to RYGB (p = 0.001).

An inherent limitation of our study is the lack of randomization. Randomization of patients is largely discouraged and often outright rejected in Germany due to strict ethical standards.

Furthermore, the study has a 95% follow-up loss rate, which is not subject to variation by the treating clinics and surgeons. Consequently, we can only characterize the outcomes of the two procedures based on the results obtained from the available data. Therefore, the conclusions drawn from the existing body of literature should be taken into account in conjunction with the recommendations of this study, and not in isolation.

Despite these limitations, our aim is to use sophisticated statistical analysis to bring existing registry data up to the standard of a randomized trial. Based on our assessment, the results of our study are comparable to those observed in international randomized trials.

## Conclusion

The primary aim of this study was to ensure the quality of bariatric surgery, to evaluate the potential hazards associated with the procedure, and define patient selection criteria for both SG and RYGB through data analysis. The study revealed significant differences in the prevalence of GERD, aHTN, and OSAS among the comorbidities studied. The incidence of newly developed postoperative reflux within five years was three times higher after SG compared to RYGB. Remission of aHTN, OSAS, IDDM, and GERD was significantly greater after RYGB than after SG.

The results of the study indicate that the RYGB procedure has superior performance in several categories, suggesting its superiority over SG.

The literature indicates that SG is a reliable and widely used bariatric surgical technique known for its straightforward technical configuration and impeccable safety record. Therefore, given the advantages of RYGB over SG in the present study, it is imperative to carefully consider the merits of each bariatric procedure in order to achieve the most favorable expected outcomes from bariatric surgery. Therefore, when evaluating the acceptability of the procedure, additional factors such as patient consent, preoperative risk factors, and surgeon expertise should be considered in addition to the outcomes of the two procedures.

In addition to the specific procedure itself, bariatric surgery typically requires a comprehensive multidisciplinary therapeutic approach, including postoperative patient care.

## Data Availability

No datasets were generated or analysed during the current study.
